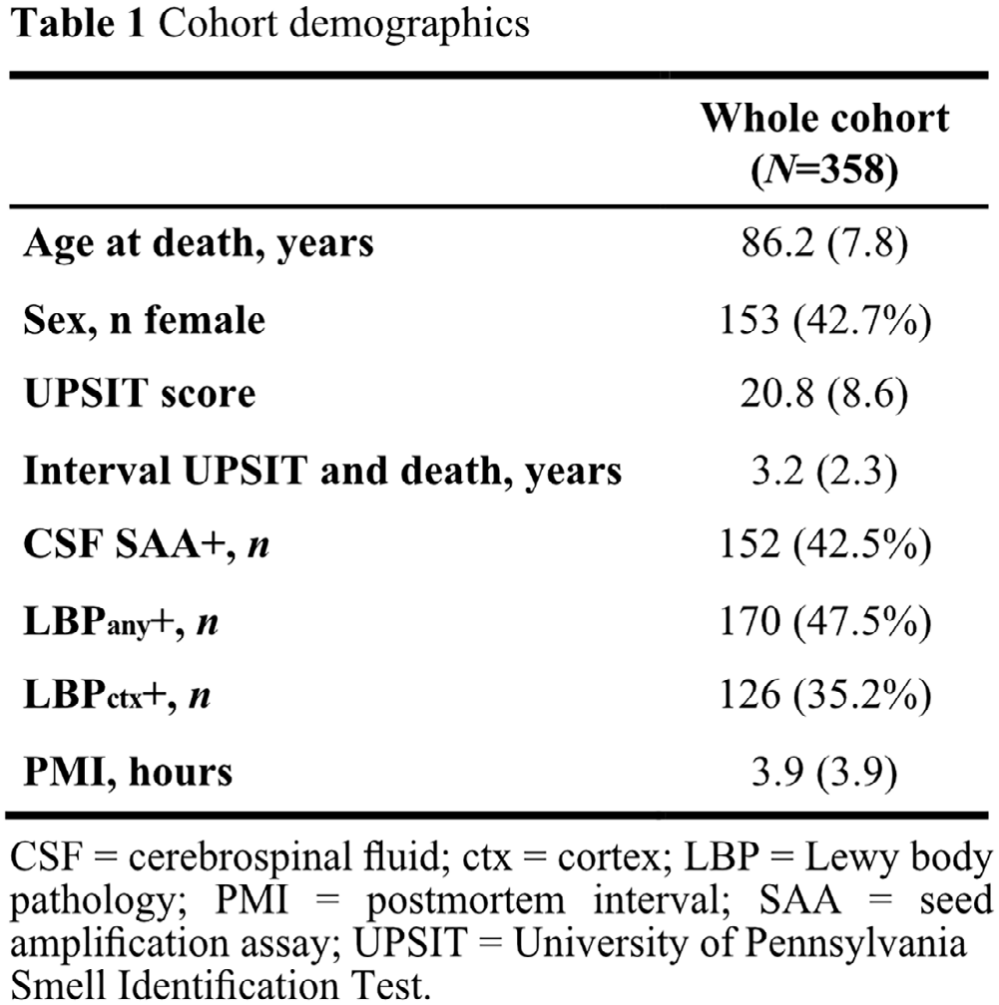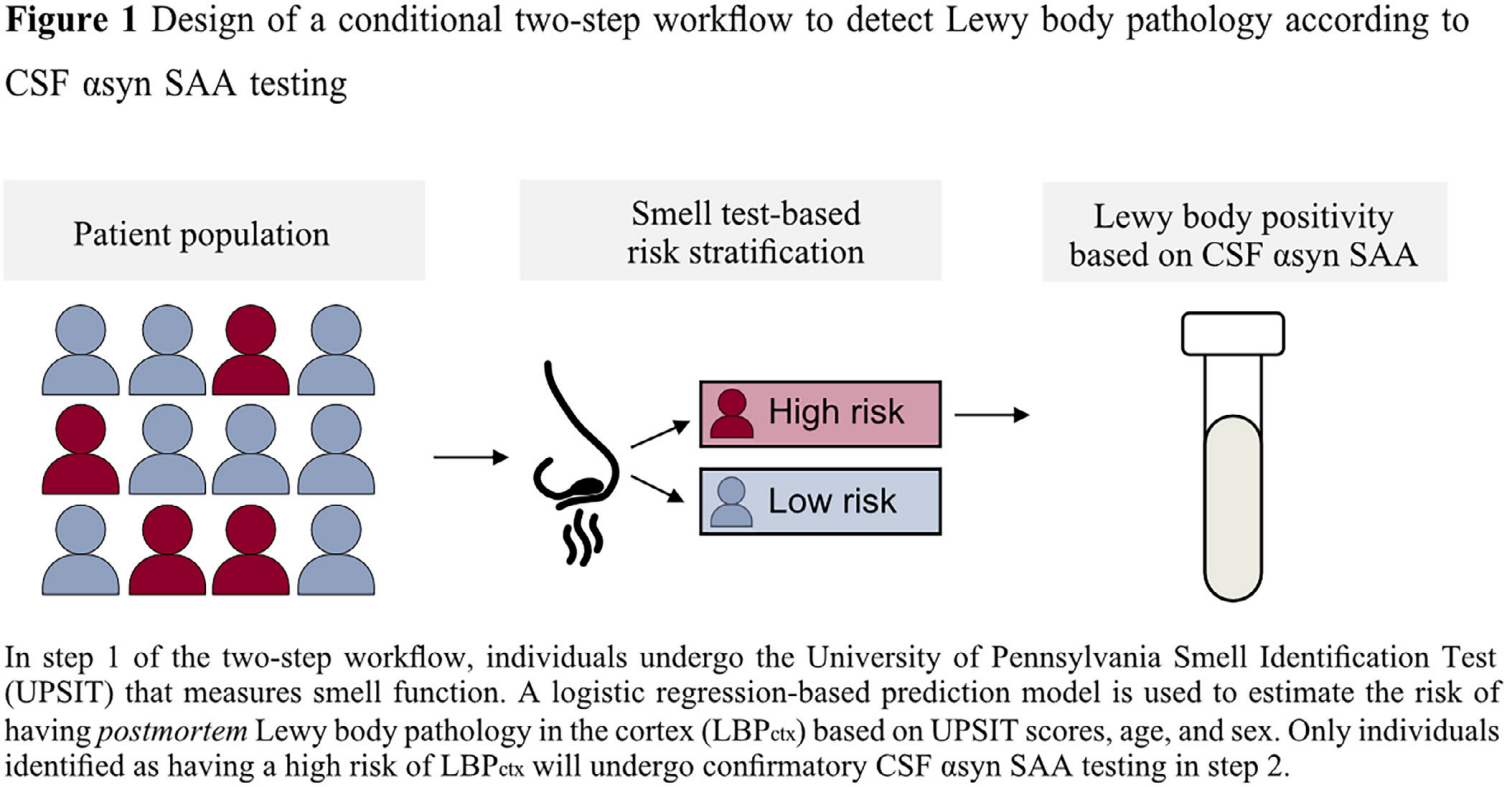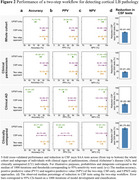# A conditional two‐step approach for detecting Lewy Body pathology: smell function testing and α‐synuclein seed amplification

**DOI:** 10.1002/alz70856_102894

**Published:** 2025-12-26

**Authors:** Sophie E. Mastenbroek, Lyduine E. Collij, Jacob W. Vogel, Serena Caldera, Geidy E Serrano, Charles Adler, Claudia Marina Vargiu, Sebastian Palmqvist, Frederik Barkhof, Piero Parchi, Thomas G Beach, Rik Ossenkoppele, Oskar Hansson

**Affiliations:** ^1^ Amsterdam Neuroscience, Brain Imaging, Amsterdam, Netherlands; ^2^ Clinical Memory Research Unit, Department of Clinical Sciences Malmö, Faculty of Medicine, Lund University, Lund, Sweden; ^3^ Department of Radiology and Nuclear Medicine, Vrije Universiteit Amsterdam, Amsterdam University Medical Center, location VUmc, Amsterdam, Netherlands; ^4^ Department of Clinical Sciences Malmö, SciLifeLab, Lund University, Lund, Sweden; ^5^ IRCCS Istituto delle Scienze Neurologiche di Bologna, Bologna, Italy; ^6^ Banner Sun Health Research Institute, Sun City, AZ, USA; ^7^ Parkinson's Disease and Movement Disorders Center, Mayo Clinic, Scottsdale, AZ, USA; ^8^ Memory Clinic, Skåne University Hospital, Malmö, Skåne, Sweden; ^9^ Institutes of Neurology & Healthcare Engineering, University College London, London, United Kingdom; ^10^ University of Bologna, Bologna, Italy; ^11^ Amsterdam Neuroscience, Neurodegeneration., Amsterdam, Netherlands; ^12^ Alzheimer Center Amsterdam, Neurology, Vrije Universiteit Amsterdam, Amsterdam UMC location VUmc, Amsterdam, Netherlands; ^13^ Clinical Memory Research Unit, Department of Clinical Sciences Malmö, Faculty of Medicine, Lund University, Sweden, Lund, Sweden

## Abstract

**Background:**

Cerebrospinal fluid (CSF) seed amplification assays (SAAs) for detecting α‐synuclein (αsyn) seeds have recently emerged as robust *in vivo* biomarkers of Lewy body pathology (LBP). However, CSF sampling is invasive, costly, and time‐consuming, limiting its widespread use. We aimed to develop and evaluate a neuropathologically‐validated two‐step workflow, leveraging smell function and CSF αsyn SAA to accurately determine *postmortem* LBP status. This approach could reduce the number of confirmatory lumbar punctures needed (Figure 1).

**Methods:**

The study included 358 individuals from the Arizona Study of Aging and Neurodegenerative Disorders with *antemortem* smell testing, CSF αsyn SAA results, and *postmortem* neuropathological assessments of regional LBP burden. Step‐1 of the two‐step workflow involved a risk‐stratification model predicting *postmortem* cortical LBP‐positivity (LBP_ctx_, defined as at least mild pathology in any cortical region) using logistic regression models with University of Pennsylvania Smell Identification Test (UPSIT) scores (smell testing), age, and sex as predictors. In step‐2, confirmatory CSF αsyn SAA testing was applied only to participants identified as high‐risk in step‐1. Workflow performance ‐ accuracy, positive predictive value (PPV), negative predictive value (NPV), and reduction in CSF testing ‐ was evaluated in (i) the entire study cohort; (ii) patients with clinical parkinsonism; (iii) patients with an Alzheimer's disease (AD) clinical syndrome; and (iv) clinically unimpaired (CU) individuals.

**Results:**

Participants had a mean age at death of 86.2±7.8 years, 42.6% were female, 35.2% had LBP_ctx_, and the average time between UPSIT and death was 3.2±2.3 years (Table 1). Using a 95% sensitivity cut‐off for the UPSIT‐based algorithm, the two‐step workflow achieved high accuracy in identifying LBP_ctx_ (whole cohort=94%; clinical parkinsonism=95%; clinical AD=94%; CU=93%; Figure 2a), while reducing CSF testing (whole cohort=‐43%; clinical parkinsonism=‐23%; clinical AD=‐35%; and CU=‐80%; Figure 2d). PPVs ranged from 75‐96% and were highest in the clinical parkinsonism subgroup (96%) where LBP_ctx_+ was highest (62.7%). NPVs ranged between 95‐98% and were highest in the clinical AD subgroup (98%) (Figure 2b‐c). The two‐step approach reached accuracies similar to using CSF tests in all participants.

**Conclusions:**

Implementing a two‐step workflow in different clinical scenarios may reduce invasive testing with CSF, minimizing the burden for individuals and costs for healthcare providers.